# Estimating the occurrence of primary ubiquinone deficiency by analysis of large-scale sequencing data

**DOI:** 10.1038/s41598-017-17564-y

**Published:** 2017-12-18

**Authors:** Bryan G. Hughes, Paul M. Harrison, Siegfried Hekimi

**Affiliations:** Department of Biology, McGill University, Montreal, Canada

## Abstract

Primary ubiquinone (UQ) deficiency is an important subset of mitochondrial disease that is caused by mutations in UQ biosynthesis genes. To guide therapeutic efforts we sought to estimate the number of individuals who are born with pathogenic variants likely to cause this disorder. We used the NCBI ClinVar database and literature reviews to identify pathogenic genetic variants that have been shown to cause primary UQ deficiency, and used the gnomAD database of full genome or exome sequences to estimate the frequency of both homozygous and compound heterozygotes within seven genetically-defined populations. We used known population sizes to estimate the number of afflicted individuals in these populations and in the mixed population of the USA. We then performed the same analysis on predicted pathogenic loss-of-function and missense variants that we identified in gnomAD. When including only known pathogenic variants, our analysis predicts 1,665 affected individuals worldwide and 192 in the USA. Adding predicted pathogenic variants, our estimate grows to 123,789 worldwide and 1,462 in the USA. This analysis predicts that there are many undiagnosed cases of primary UQ deficiency, and that a large proportion of these will be in developing regions of the world.

## Introduction

Mitochondrial disease is a complex and heterogeneous collection of disorders that can result in death or prolonged disability. The prevalence of these disorders could be as high as 1 in 4,300 individuals, which makes it one of the most common forms of inherited illness^1,2^. There is no general cure for these disorders but the most widespread treatments are vitamin and nutritional supplementation, most commonly with L-carnitine, creatine and ubiquinone (UQ; a.k.a. Coenzyme Q), despite the fact that there is little evidence supporting their effectiveness^3,4^.

UQ is a redox-active lipid-like molecule that plays a number of critical roles in biological membranes. Its best characterized role is as a key electron carrier of the mitochondrial electron transport chain^5^. UQ is also a co-factor in a number of other enzymatic processes as well as a potential membrane antioxidant. The rationale for generalized UQ supplementation in mitochondrial disease is thus the hope that it might support mitochondrial function, and that its antioxidant function could ameliorate any increase in oxidative stress. Furthermore, UQ deficiency secondary to other mitochondrial defects is observed in a substantial subset of mitochondrial disease patients^6^.

There is, however, one patient population that could directly benefit from effective UQ supplementation: individuals suffering from primary UQ deficiency due to mutations in genes required for UQ biosynthesis. Although such patients have been much discussed^7–9^, we are not aware of any formal attempt to estimate the prevalence of primary UQ deficiency. At this point, approximately 70 patients have been described in the published literature, and it has been informally estimated that their prevalence may be less than 1 in 100,000^8^. Despite clear genetic evidence that UQ deficiency is the primary cause in these patients, UQ supplementation has not met with consistent success, possibly due to poor bioavailability of the highly lipophilic UQ molecule^10,11^. A better understanding of the possible prevalence of this disorder would help guide decisions regarding investigations into novel UQ formulations or potential drugs which could modulate the UQ biosynthesis pathway.

UQ is composed of a redox-active benzoquinone ring with a lipid tail consisting of a species-specific number of isoprenoid sub-units (ten in humans). Although UQ biosynthesis has been most extensively studied in yeast, human homologues of the critical genes have been identified^7–9^. Thirteen yeast genes are required for UQ biosynthesis (COQ1 – COQ11, YAH1, ARH1). In brief, COQ1 (or the human homologues PDSS1 and PDSS2 acting as a hetero-tetramer) assembles an isoprenoid tail from precursors produced by the mevalonate pathway. COQ2 joins this isoprenoid tail to a tyrosine-derived benzoquinone ring precursor, and COQ3, COQ5, COQ6 and COQ7 are responsible for various methylation and hydroxylation reactions affecting the benzoquinone ring. COQ8 appears to play a regulatory role by modulating phosphorylation of COQ3, COQ5 and COQ7. COQ8 has two human homologues, COQ8A (also known as ADCK3 or CABC1) and COQ8B (ADCK4), both of which can independently result in UQ deficiency^12,13^. The roles of COQ4 and COQ9 are not well defined, although COQ4 appears to play a role in the assembly of COQ2 – COQ7 into a complex and COQ9 is required for COQ7 function. ARH1 (human homologue FDX1L) and YAH1 (FDXR) transfer electrons to COQ6, while also participating in other pathways. There are two modification steps of the UQ benzoquinone ring that have yet to be assigned an enzyme.

To date, pathogenic variants in nine of these proteins (PDSS1, PDSS2, COQ2, COQ4, COQ6, COQ7, COQ8A, COQ8B and COQ9) have been shown to cause UQ deficiency in human patients^7,9^. We sought to leverage the recent availability of exome or genome sequences of very large numbers of individuals in order to estimate the frequency of known pathogenic variants in these genes. We used the NCBI ClinVar database^14^ and conducted a literature search to identify variants in the known UQ biosynthesis genes that result in illness and UQ deficiency. The gnomAD exome and genome database^15^, with sequences for almost 138,632 individuals divided into seven genetically-distinct populations, was used to estimate the frequencies of these variants. Using these frequencies, we estimated the birth prevalence of individuals homozygous or compound heterozygous for known or predicted pathogenic genetic variants for primary UQ deficiency (assuming Hardy-Weinberg equilibria) on a population-by-population basis and used known population sizes and distributions to estimate the actual numbers of afflicted individuals due to each variant world-wide, as well as in a population with the particular size and mix of the USA. Importantly, the calculation of the number of afflicted individuals on a per-variant, per-population, basis eliminates a potential confounding factor when working with large numbers of variants present at very low frequencies – namely, that many individual variants may be too rare to result in any homozygous or compound heterozygous individuals, and the traditional method of summing these frequencies could yield frequencies high enough to artificially suggest that individuals are affected.

It is likely that many pathogenic variants simply have not been clinically documented at this relatively early stage in our awareness of primary UQ deficiency. To account for this, we also estimated the number of individuals who would be homozygous or compound heterozygous for variants observed in gnomAD but that have not yet been observed in the clinic, focusing on predicted loss-of-function (LoF) or pathogenic missense mutations.

There are many challenges to making estimates of this nature. For example, it is not possible to conclusively determine the pathogenicity of missense variants based on sequence information alone. We attempt to address this by conservatively included only those variants independently predicted to be pathogenic by two separate bioinformatic algorithms (see Methods). There is also extreme variability in severity of primary UQ deficiency, ranging from neonatal lethality (with mouse studies suggesting that embryonic lethality is a possible outcome for null alleles for some genes^16–19^) to mild disease that becomes apparent only in later decades of life. This makes accurate predictions of disease prevalence based on allelic frequencies extrapolated from public databases of genomic variants challenging, which is why our results are best interpreted as birth prevalence of individuals homozygous or compound heterozygous for variants likely to cause disease. Actual disease prevalence would be expected to diverge from these estimates. We discuss these issues in greater detail below.

We found that the carrier frequencies for most previously identified pathogenic variants were low (averaging 1/6,420 for the populations in which they were present), and given known population sizes we estimated they would result in a total of 1,016 individuals worldwide due to homozygosity and an additional 649 due to compound heterozygosity, with a total of 192 in the USA. The addition of all predicted loss-of-function and pathogenic missense variants results in a predicted total of 123,789 individuals worldwide and 1,462 in the USA.

## Methods

### Identification of known pathogenic variants

We identified pathogenic variants of UQ biosynthesis genes (PDSS1, PDSS2, COQ2 – COQ7, COQ8A/ADCK3, COQ8B/ADCK4 and COQ9) using the NCBI ClinVar database and via PubMed literature searches. ClinVar is a public archive (https://www.ncbi.nlm.nih.gov/clinvar/) describing human genetic variants and their relationship to human health^14^. Variants are extracted from the peer-reviewed literature or directly reported by CLIA certified or ISO 1589 accredited clinical testing laboratories. Variant pathogenicity is reported by the submitter according to the ordinal scale recommended by the American College of Medical Genetics and Genomics and the Association for Molecular Pathology (“pathogenic”, “likely pathogenic”, “uncertain significance”, “likely benign” or “benign”)^20^. Note that ClinVar results cannot be used to directly estimate birth prevalence and the database does not include fields for incidence frequency. Each ClinVar entry describes a unique variant, and may be derived from multiple submissions.

We queried ClinVar (search conducted on 2017-03) for each gene (e.g., ‘COQ2[gene]’) and identified pathogenic variants using the following inclusion criteria:

(i) At least one submission describes the variant as “pathogenic” or “likely pathogenic”.

(ii) No submitter assigns a significance as “benign” or “likely benign”.

(iii) Variant only affects one gene (i.e., no multi-gene deletions or duplications).

Complete records for all variants meeting our inclusion criteria were manually reviewed, including confirming that the record matches the description in any cited studies. To ensure as complete as possible a record of known variants, we also conducted a systematic literature search via pubmed (search conducted on 2017-01), where we reviewed all clinical studies in the search results for each gene name.

### COQ2 transcript start

It was recently shown that the canonical COQ2 transcript, used by most previous studies, erroneously includes a 150 base N-terminal region that is only rarely, if ever, transcribed in humans^21^. Thus, any variant involving this region is unlikely to be pathogenic. For ease of comparability with previous studies we have retained the canonical numbering, but we have not included any variant affecting this region. For this reason, we did not consider variants such as p.Ala17Argfs (ClinVar allele ID 237155).

### Estimation of variant birth prevalence

To determine the birth prevalence of these variants we used the Genome Aggregation Database (gnomAD, http://gnomad.broadinstitute.org/), an updated version of the previously released dataset from the Exome Aggregation Consortium (ExAC)^15^. The gnomAD release includes exome or genome sequences from a total of 138,632 individuals without severe pediatric diseases. The database assigns ancestry by a principal components analysis based upon a subset of samples of known ancestry. Most sequences clustered into one of seven geographic or endogamous groups (African, Ashkenazi Jews, East Asian, Finnish, non-Finnish Europeans, Latin Americans, and South American), with the remainder (3,234) considered to be ‘other’. Hence, almost all samples included in this dataset have an ancestry that is well-defined on a genetic basis. This dataset has undergone extensive quality control measures to remove poor-quality sequences, related individuals and to flag variants of questionable reliability^15^, and assessments of variant pathogenicity are provided via the SIFT and PolyPhen2 tools.

When querying gnomAD for each of our genes of interest, variants affecting protein-coding regions were considered equivalent to known pathogenic variants if they resulted in the same change to protein structure (i.e., the same amino acid conversion, a stop codon introduced in the same location, or a frameshift resulting in the same residue changes). For variants affecting splice sites, only variants that exactly matched the nucleotide changes of the pathogenic variants were included. We only considered variants that had passed gnomAD random forest filters.

We acquired estimates of population sizes from various sources (Table S1). The population estimates summed to 6 billion, accounting for 80% of total global population. Estimates of population sizes within the USA summed to 309 million (vs. a total population of approx. 319 million). To estimate the number of affected individuals, we used the individual frequencies for each variant in a population (i.e., not on summed frequencies), and estimates for each variant for a population were rounded down to the nearest whole number.

Rates of compound heterozygosity were determined using data tables of missense and loss-of-function variants for each gene obtained from the gnomAD browser. An R script (available upon request) was written to systematically strip out undesirable variants (e.g., those affecting non-canonical transcripts) and make the multiple comparisons required. We calculated the predicted frequency of compound heterozygotes within each population in which it was possible (i.e., some variants were not observed in the same population, making compound heterozygosity for that variant impossible in that population). When determining rates of compound heterozygosity for the group of predicted loss-of-function (LoF) and pathogenic missense variants, we included known pathogenic/predicted LoF variant pairs in our calculations.

Pearson’s chi-square test of goodness of fit was calculated in Excel 14.0.7180.5002 (Microsoft, USA), and other calculations were performed in R. 95 percent confidence intervals were calculated with the exact binomial test.

### Identification of predicted pathogenic variants

To identify predicted pathogenic variants in the gnomAD database, we first excluded variants that did not pass quality-control filters and those in non-canonical transcripts (as defined by gnomAD, the canonical transcript is the longest consensus coding sequence translation with no stop codons). To identify LoF variants, we extracted those annotated as “stop gained”, “frameshift”, “splice donor” or “splice acceptor” and excluded variants which gnomAD had flagged as low-confidence LoF. To identify the missense variants that were most likely to be pathogenic, we extracted only those variants for which gnomAD reported an assessment of “probably damaging” and “deleterious” by PolyPhen2 and SIFT respectively. To reduce the risk of obtaining false positives, we excluded variants with high minor allele (MAF) frequencies. Although a MAF cut-off of 0.5% has been suggested^22^, we chose a more conservative approach, instead using the highest observed MAF in the list of “known” pathogenic variants as a threshold: thus variants with a global MAF greater than 0.019% or a MAF for any population greater than 0.31% were excluded.

### Data Availability

The datasets analyzed during the current study are available at www.ncbi.nlm.nih.gov/clinvar/ and http://gnomad.broadinstitute.org/.

## Results

Through ClinVar, we identified 552 reported genetic variants affecting UQ biosynthesis genes (for complete listing, see File S1). Of these, 143 were deletions or duplications affecting multiple genes (all 17 variants reported for COQ3 and COQ5 fell into this category), and were not considered further because the pathogenicity of these variants could potentially be related to the activity of multiple genes. Of the remainder, 315 were excluded because submitters did not assess them as pathogenic (in only one case, ClinVar variation 3645, COQ8A p.Phe331=, were there both pathogenic and benign interpretations – in this case, a MAF of 1.57% supports the benign interpretation). Thirteen of those remaining were subsequently excluded because close inspection of the records and cited works revealed a number of problems, including single-copy variants not consistent with the typically recessive nature of UQ deficiencies (4 records), duplicate records (4), risk factors mis-categorized as causative pathogenic variants (2), an incomplete ClinVar entry (1), a multi-variant haplotype not testable in gnomAD (1), reliance on a secondary, unreferenced, source (1), and one variant present in the untranscribed N-terminal region of COQ2 (see Methods).

Of the remaining 80 records, the majority (49) had been extracted from the peer-reviewed literature, 22 were from the genetic testing company GeneDx (MD, USA), with the remainder from 6 other testing labs (see Table S2 for detailed information). GeneDx and five other testing labs provided detailed assertion criteria for the determination of variant pathogenicity, all adhering to established standards.

To account for the possibility that not all known pathogenic variants are included in ClinVar, we carried out an independent review of the literature, identifying 18 additional pathogenic variants: 2 affecting COQ2, 3 COQ4, 1 COQ6, 9 COQ8A, and 2 COQ8B (see Table S2 for literature references).

In total, we identified 97 pathogenic variants. Of these, 57 resulted in a single residue substitution, 21 in frameshifts, 10 premature stop codons, 7 variants altering splice-site donor or acceptor regions in ways predicted to be pathogenic, and 3 single-residue indels (see Table S2 for a complete listing of all identified known pathogenic variants). COQ8A was most frequently affected, with 40 variants.

To better understand the birth prevalence of these variants we queried the gnomAD exome and genome database. We found 441 carriers, with 49 of 97 pathogenic variants present (Table 1). No variants were present in homozygous form, all missense variants were predicted to be damaging by PolyPhen2, SIFT, or both, and all premature stop, frameshift or splice site-disrupting variants were predicted to be high-confidence loss-of-function. All of these findings are fully consistent with the reported pathogenic nature of these variants. Global allele frequencies ranged from 4.1 × 10^−6^ to 1.7 × 10^−4^, yielding a combined frequency of 1.76 × 10^−3^, implying that 1/321,368 individuals will be homozygous for pathogenic variants at birth.

**Table 1:** Known pathogenic variants from ClinVar or literature review that are represented in gnomAD sequence database.

Gene	Consequence	Source^1^		Clinical Sign.^2^	Predicted effect on protein function			Carriers	Allele Freq.
		Lit.	Lab		PolyPhen2	SIFT	LoF		
PDSS1	p.Asp308Glu	1	0	P	prob. damaging	deleterious		1	4.07E-06
PDSS2	p.His92Pro	0	1	LP	prob. damaging	deleterious		11	3.97E-05
	p.Gln322*	1	0	P	N/A	N/A	high-conf.	1	4.07E-06
	p.Ser382Leu	1	0	P	prob. damaging	deleterious		7	2.84E-05
COQ2	p.Ser146Asn	1	0	P	prob. damaging	deleterious		4	1.67E-05
	p.Arg197His	1	0	P	prob. damaging	deleterious		2	8.14E-06
	p.Asn228Ser	1	0	P	benign	deleterious		32	1.16E-04
	p.Leu234Argfs*14	1^&^	0	N/A	N/A	N/A	high-conf.	1	4.19E-06
	p.Tyr297Cys	1	0	P	prob. damaging	deleterious		1	4.11E-06
	p.Thr317Met	0	1	P	prob. damaging	deleterious		5	2.72E-05
	p.Gly390Ala	1^&^	0	N/A	prob. damaging	deleterious		6	2.45E-05
	p.Asn401Ilefs*15	1	0	P	N/A	N/A	high-conf.	3	1.23E-05
COQ4	p.Pro64Ser	1	0	P	prob. damaging	deleterious		2	9.07E-06
	p.Asp68His	1	0	P	prob. damaging	deleterious		10	4.22E-05
	p.Pro119Leu	0	1	LP	prob. damaging	deleterious		3	1.22E-05
	p.Arg145Gly	1	0	P	prob. damaging	tolerated		1	4.75E-06
	p.Arg240Cys	2	1	P	prob. damaging	deleterious		47	1.72E-04
COQ6	p.Pro261Leu	1^&^	0	N/A	prob. damaging	deleterious		19	6.86E-05
	p.Asn380Ser	0	1	LP	prob. damaging	deleterious		6	2.16E-05
COQ8A	c.589–3 C > G	1^&^	0	N/A	N/A	N/A	conf.^3^	1	3.23E-05
	p.Arg213Trp	1	0	P	prob. damaging	damaging		2	6.46E-05
	p.Arg213Glnfs*71	0	1	P	N/A	N/A	high-conf.	1	4.07E-06
	p.Arg271Cys	2	1	LP;US	prob. damaging	deleterious		29	1.08E-04
	p.Leu277Pro	0	1	LP	pos. damaging	deleterious		1	4.49E-06
	p.Arg299Trp	1^&^	0	N/A	prob. damaging	deleterious		8	2.89E-05
	p.Ala304Thr	1^&^	0	N/A	prob. damaging	deleterious		4	1.63E-05
	p.Ala304Val	0	1	LP	prob. damaging	deleterious		11	3.97E-05
	p.Arg348Ter	0	1	P	N/A	N/A	high-conf.	10	4.04E-05
	p.Leu379*	1	0	N/A	N/A	N/A	high-conf.	1	4.06E-06
	p.Arg410*	0	1	N/A	N/A	N/A	high-conf.	5	1.82E-05
	p.Thr445Argfs*52	0	1	P	N/A	N/A	high-conf.	1	4.07E-06
	p.Thr511Met	0	1	LP	pos. damaging	deleterious		13	4.69E-05
	p.Gly549Ser	1	0	P	prob. damaging	deleterious		35	1.27E-04
	p.Glu551Lys	1	0	P	prob. damaging	deleterious		5	1.81E-05
	p.Met555Ile	1	0	LP	pos. damaging	deleterious		41	1.49E-04
	p.Glu568*	0	1	P	N/A	N/A	high-conf.	1	4.07E-06
	p.Ser582Glufs*148	0	1	P	N/A	N/A	high-conf.	4	1.63E-05
	p.Thr584del	1	1	P	N/A	N/A	N/A^4^	30	1.22E-04
	p.Pro602Arg	1^&^	0	N/A	prob. damaging	deleterious		9	3.25E-05
	p.Pro602Gln	0	1	P	prob. damaging	deleterious		10	4.06E-05
	p.Ser616Leufs*114	0	1	P	N/A	N/A	high-conf.	16	5.77E-05
COQ8B	p.Phe215Leufs*14	1	0	N/A	N/A	N/A	high-conf.	10	3.67E-05
	p.Asp286Gly	1	0	P	pos. damaging	deleterious		4	1.45E-05
	p.Arg320Trp	1	0	P	prob. damaging	deleterious		5	2.03E-05
	p.His400Glnfs*11	1	0	P	N/A	N/A	high-conf.	1	4.06E-06
	p.Glu447Glyfs*10	1^&^	0	N/A	N/A	N/A	high-conf.	6	2.48E-05
	p.Glu483*	1	0	P	N/A	N/A	high-conf.	11	4.83E-05
COQ9	c.521 + 1delG	1	1	P	N/A	N/A	high-conf.	1	4.08E-06
	p.Arg244*	1	0	P	N/A	N/A	high-conf.	3	1.22E-05
Sum:								441	1.76E-03

See Table S2 for more information on all known pathogenic variants.

^1^Number of reports in the peer-reviewed literature (^&^: sources not included in ClinVar) or directly submitted by testing labs.

^2^Clinical significance, as stated in ClinVar record: Pathogenic, Likely Pathogenic, Uncertain Significance.

^3^Acceptor splice site mutation c.589–3 C > G demonstrated by *in vitro* model to result in non-functional product p.Leu197Valfs*20.

^4^In-frame deletion; mutant ADCK3 failed to rescue growth in ADCK3-null yeast.

Through casual observation it was apparent that several of the known pathogenic variants were not distributed evenly within the different populations. For example, the COQ8A p.Met555Ile variant was observed in 39 European or Finnish individuals, but in no other population, and the COQ8B p.Glu483* variant was observed in 10 individuals from South Asia but only in 1 European, despite the almost 4-fold greater number of European alleles genotyped. Indeed, the six variants with the greatest numbers of carriers had frequencies that were distributed unevenly between populations (Pearson’s chi-squared 24.3 to 537.5, p < 0.001) (Figure S1 - statistically significant differences were rarer among the variants with lower allele counts, potentially due to the decreased statistical power inherent in a lower sample size). Because of this unevenness, subsequent analysis was conducted on a population-by-population basis.

Each pathogenic variant was observed in an average of 1.9 populations (not counting ‘Other’), with an average allele frequency of 1.56 × 10^–4^ (Table 2). Combined estimates of Hardy-Weinberg homozygosity for all variants for each of the 7 populations averaged 1/5,492,983, ranging from 1/12,021,014 (Latin Americans) to 1/60,113 (Ashkenazi Jews). Predicted homozygous frequency for individual variants averaged 1/5.4 M, with the variant found at the greatest frequency being COQ4 p.Arg240Cys (with a 1/162 carrier frequency among Ashkenazi Jews which would result in the birth of homozygotes at a frequency of 1/104,733). With an estimated worldwide population of 10 M, this would imply 95 afflicted Ashkenazi Jews susceptible to UQ deficiency due to homozygosity for this one variant alone. Considering all variants across all populations, we can predict 1,016 homozygous-at-birth individuals globally, or 122 in the USA (Table 2).

**Table 2:** Population breakdown and predicted prevalence of afflicted individuals for known pathogenic variants present in the gnomAD database.

Gene	Consequence	Pop^n1^	Allele Frequency		Predicted Homozygous (with 95% CIs)^2^		USA^4^
			Actual	Freq.	Frequency	Global^3^	
PDSS1	p.Asp308Glu	Ash	1/9,834	1.02E-04	1.03E-8 (6.63E-12 – 3.21E-7)	<1 (<1–3)	<1 (<1–1)
PDSS2	p.His92Pro	Afr	2/23,986	8.34E-05	6.95E-9 (1.02E-10–9.07E-8)	8 (<1–107)	<1 (<1–3)
		Eur	9/126,452	7.12E-05	5.07E-9 (1.06E-9–1.83E-8)	3 (<1–13)	1 (<1–3)
	p.Gln322*	Eur	1/111,562	8.96E-06	8.03E-11 (5.15E-14–2.49E-9)	<1 (<1 – 1)	<1 (<1 –<1)
	p.Ser382Leu	EAs	1/17,240	5.80E-05	3.36E-9 (2.16E-12 – 1.04E-7)	5 (<1–168)	<1 (<1 –<1)
		Eur	5/111,682	4.48E-05	2.0E-9 (2.11E-10–1.09E-8)	1 (<1–7)	<1 (<1–2)
		Lat	1/33,582	2.98E-05	8.87E-10 (5.68E-13–2.75E-8)	<1 (<1–17)	<1 (<1–1)
COQ2	p.Ser146Asn	Eur	4/107,234	3.73E-05	1.39E-9 (1.03E-10–9.12E-9)	1 (<1–6)	<1 (<1–1)
	p.Arg197His	EAs	1/17,222	5.81E-05	3.37E-9 (2.16E-12–1.05E-7)	5 (<1–168)	<1 (<1 –<1)
		Eur	1/111,452	8.97E-06	8.05E-11 (5.16E-14–2.50E-9)	<1 (<1–1)	<1 (<1 –<1)
	p.Asn228Ser	Eur	30/126,254	2.38E-04	5.65E-8 (2.57E-8–1.15E-7)	41 (18–84)	11 (5–22)
		Afr	1/23,990	4.17E-05	1.74E-9 (1.11E-12–5.39E-8)	2 (<1–63)	<1 (<1–2)
		Lat	1/34,214	2.92E-05	8.54E-10 (5.48E-13–2.65E-8)	<1 (<1–16)	<1 (<1–1)
	p.Leu234Argfs*14	Eur	1/108,122	9.25E-06	8.55E-11 (5.48E-14–2.66E-9)	<1 (<1–1)	<1 (<1 –<1)
	p.Tyr297Cys	SAs	1/30,338	3.30E-05	1.09E-9 (6.96E-13–3.37E-8)	1 (<1–61)	<1 (<1 –<1)
	p.Thr317Met	EAs	1/11,902	8.40E-05	7.06E-9 (4.52E-12–2.19E-7)	11 (<1–353)	<1 (<1–1)
		Afr	1/16,630	6.01E-05	3.62E-9 (2.32E-12–1.12E-7)	4 (<1–133)	<1 (<1–4)
		Eur	3/73,824	4.06E-05	1.65E-9 (7.02E-11–1.41E-8)	1 (<1–10)	<1 (<1–2)
	p.Gly390Ala	Lat	5/33,178	1.51E-04	2.27E-8 (2.39E-9–1.24E-7)	14 (1–78)	1 (<1–6)
		Eur	1/111,216	8.99E-06	8.08E-11 (5.18E-14–2.51E-9)	<1 (<1–1)	<1 (<1 –<1)
	p.Asn401Ilefs*15	Eur	3/110,906	2.70E-05	7.32E-10 (3.11E-11–6.25E-9)	<1 (<1–4)	<1 (<1–1)
COQ4	p.Pro64Ser	Eur	2/97,392	2.05E-05	4.22E-10 (6.18E-12–5.50E-9)	<1 (<1–4)	<1 (<1–1)
	p.Asp68His	Lat	3/28,866	1.04E-04	1.08E-8 (4.59E-10–9.22E-8)	6 (<1–58)	<1 (<1–5)
		Eur	6/107,660	5.57E-05	3.11E-9 (4.18E-10–1.47E-8)	2 (<1–10)	<1 (<1–2)
		Oth	1/5,584	1.79E-04	—	—	—
	p.Pro119Leu	Afr	3/15,302	1.96E-04	3.84E-8 (1.63E-9–3.28E-7)	45 (1–389)	1 (<1–13)
	p.Arg145Gly	SAs	1/26,964	3.71E-05	1.38E-9 (8.82E-13–4.27E-8)	2 (<1–77)	<1 (<1 –<1)
	p.Arg240Cys	Ash	31/10,032	3.09E-03	9.55E-6 (4.41E-6–1.92E-5)	95 (44–192)	48 (22–98)
		Eur	9/124,924	7.20E-05	5.19E-9 (1.09E-9–1.87E-8)	3 (<1–13)	1 (<1–3)
		Lat	2/33,836	5.91E-05	3.49E-9 (5.12E-11–4.56E-8)	2 (<1–28)	<1 (<1–2)
		Oth	5/6,362	7.86E-04	—	—	—
COQ6	p.Pro261Leu	Afr	3/24,026	1.25E-04	1.56E-8 (6.63E-10–1.33E-7)	18 (<1–157)	<1 (<1–5)
		EAs	2/18,870	1.06E-04	1.12E-8 (1.65E-10–1.47E-7)	18 (<1–236)	<1 (<1 –<1)
		Eur	13/126,674	1.03E-04	1.05E-8 (2.99E-9–3.08E-8)	7 (2–22)	2 (<1–6)
		SAs	1/30,782	3.25E-05	1.06E-9 (6.76E-13–3.28E-8)	1 (<1–59)	<1 (<1 –<1)
	p.Asn380Ser	Afr	5/24,036	2.08E-04	4.33E-8 (4.56E-9–2.36E-7)	51 (5–279)	1 (<1–9)
		EAs	1/18,870	5.30E-05	2.81E-9 (1.80E-12–8.72E-8)	4 (<1–140)	<1 (<1 –<1)
COQ8A	c.589–3 C > G	Eur	1/15,000	6.67E-05	4.44E-9 (2.85E-12–1.38E-7)	3 (<1–101)	<1 (<1–27)
	p.Arg213Trp	Eur	2/14,998	1.33E-04	1.78E-8 (2.61E-10–2.32E-7)	13 (<1–169)	3 (<1–45)
	p.Arg213Glnfs*71	Eur	1/111,444	8.97E-06	8.05E-11 (5.16E-14–2.50E-9)	<1 (<1–1)	<1 (<1 –<1)
	p.Arg271Cys	Fin	20/25,144	7.95E-04	6.33E-7 (2.36E-7–1.51E-6)	3 (1–8)	<1 (<1 –<1)
		Eur	7/122,222	5.73E-05	3.28E-9 (5.30E-10–1.39E-8)	2 (<1–10)	<1 (<1–2)
		Oth	2/6,304	3.17E-04	—	—	—
	p.Leu277Pro	Eur	1/99,064	1.01E-05	1.02E-10 (6.53E-14–3.16E-9)	<1 (<1–2)	<1 (<1 –<1)
	p.Arg299Trp	Lat	3/34,414	8.72E-05	7.60E-9 (3.23E-10–6.49E-8)	4 (<1–41)	<1 (<1–3)
		Eur	5/126,472	3.95E-05	1.56E-9 (1.65E-10–8.51E-9)	1 (<1–6)	<1 (<1–1)
	p.Ala304Thr	Ash	1/9,836	1.02E-04	1.03E-8 (6.63E-12–3.21E-7)	<1 (<1–3)	<1 (<1–1)
		Afr	1/15,270	6.55E-05	4.29E-9 (2.75E-12–1.33E-7)	5 (<1–157)	<1 (<1–5)
		SAs	1/30,782	3.25E-05	1.06E-9 (6.76E-13–3.28E-8)	1 (<1–59)	<1 (<1 –<1)
		Eur	1/111,486	8.97E-06	8.05E-11 (5.16E-14–2.50E-9)	<1 (<1–1)	<1 (<1 –<1)
	p.Ala304Val	Eur	10/126,438	7.91E-05	6.26E-9 (1.44E-9–2.12E-8)	4 (1–15)	1 (<1–4)
		Oth	1/6,456	1.55E-04	—	—	—
	p.Arg348*	Lat	3/31,814	9.43E-05	8.89E-9 (3.78E-10–7.59E-8)	5 (<1–48)	<1 (<1–4)
		Eur	7/110,946	6.31E-05	3.98E-9 (6.44E-10–1.69E-8)	2 (<1–12)	<1 (<1–3)
	p.Leu379*	Eur	1/111,658	8.96E-06	8.02E-11 (5.14E-14–2.49E-9)	<1 (<1–1)	<1 (<1 –<1)
	p.Arg410*	EAs	2/18,820	1.06E-04	1.13E-8 (1.66E-10–1.47E-7)	18 (<1–237)	<1 (<1 –<1)
		SAs	1/30,750	3.25E-05	1.06E-9 (6.78E-13–3.28E-8)	1 (<1–59)	<1 (<1 –<1)
		Eur	2/125,096	1.60E-05	2.56E-10 (3.75E-12–3.34E-9)	<1 (<1–2)	<1 (<1 –<1)
	p.Thr445Argfs*52	Lat	1/33,562	2.98E-05	8.88E-10 (5.69E-13–2.76E-8)	<1 (<1–17)	<1 (<1–1)
	p.Thr511Met	Lat	5/34,420	1.45E-04	2.11E-8 (2.22E-9–1.15E-7)	13 (1–72)	1 (<1–6)
		Ash	1/10,150	9.85E-05	9.71E-9 (6.22E-12–3.01E-7)	<1 (<1–3)	<1 (<1–1)
		Eur	7/126,688	5.53E-05	3.05E-9 (4.94E-10–1.30E-8)	2 (<1–9)	<1 (<1–2)
	p.Gly549Ser	Eur	30/126,484	2.37E-04	5.63E-8 (2.56E-8–1.15E-7)	41 (18–83)	11 (5–22)
		Fin	2/25,596	7.81E-05	6.11E-9 (8.95E-11–7.97E-8)	<1 (<1 –<1)	<1 (<1 –<1)
		Afr	1/23,988	4.17E-05	1.74E-9 (1.11E-12–5.39E-8)	2 (<1–63)	<1 (<1–2)
		Lat	1/34,410	2.91E-05	8.45E-10 (5.41E-13–2.62E-8)	<1 (<1–16)	<1 (<1–1)
		Oth	1/6,456	1.55E-04	—	—	—
	p.Glu551Lys	Eur	4/126,382	3.17E-05	1.0E-9 (7.44E-11–6.57E-9)	<1 (<1–4)	<1 (<1–1)
		Oth	1/6,454	1.55E-04	—	—	—
	p.Met555Ile	Fin	18/24,584	7.32E-04	5.36E-7 (1.88E-7–1.34E-6)	2 (1–7)	<1 (<1 –<1)
		Eur	21/126,250	1.66E-04	2.77E-8 (1.06E-8–6.46E-8)	20 (7–47)	5 (2–12)
		Oth	2/6,446	3.10E-04	—	—	—
	p.Glu568*	Eur	1/111,654	8.96E-06	8.02E-11 (5.14E-14–2.49E-9)	<1 (<1–1)	<1 (<1 –<1)
	p.Ser582Glufs*148	Lat	1/33,580	2.98E-05	8.87E-10 (5.68E-13–2.75E-8)	<1 (<1–17)	<1 (<1–1)
		Eur	3/111,712	2.69E-05	7.21E-10 (3.07E-11–6.16E-9)	<1 (<1–4)	<1 (<1–1)
	p.Thr584del	Ash	26/9,848	2.64E-03	6.97E-6 (2.98E-6–1.49E-5)	69 (29–149)	35 (15–76)
		Eur	4/111,702	3.58E-05	1.28E-9 (9.52E-11–8.41E-9)	<1 (<1–6)	<1 (<1–1)
	p.Pro602Arg	Ash	3/10,148	2.96E-04	8.74E-8 (3.72E-9–7.46E-7)	<1 (<1–7)	<1 (<1–3)
		Lat	2/34,420	5.81E-05	3.38E-9 (4.95E-11–4.41E-8)	2 (<1–27)	<1 (<1–2)
		SAs	1/30,782	3.25E-05	1.06E-9 (6.76E-13–3.28E-8)	1 (<1–59)	<1 (<1 –<1)
		Eur	2/126,676	1.58E-05	2.49E-10 (3.66E-12–3.25E-9)	<1 (<1–2)	<1 (<1 –<1)
		Oth	1/6,464	1.55E-04	—	—	—
	p.Pro602Gln	SAs	2/30,782	6.50E-05	4.22E-9 (6.19E-11–5.51E-8)	7 (<1–100)	<1 (<1 –<1)
		Eur	7/111,706	6.27E-05	3.93E-9 (6.35E-10–1.67E-8)	2 (<1–12)	<1 (<1–3)
		Oth	1/5,486	1.82E-04	—	—	—
	p.Ser616Leufs*114	EAs	6/18,866	3.18E-04	1.01E-7 (1.36E-8–4.79E-7)	163 (21–772)	<1 (<1–2)
		SAs	4/30,782	1.30E-04	1.69E-8 (1.25E-9–1.11E-7)	30 (2–201)	<1 (<1 –<1)
		Afr	1/24,020	4.16E-05	1.73E-9 (1.11E-12–5.38E-8)	2 (<1–63)	<1 (<1–2)
		Eur	5/126,684	3.95E-05	1.56E-9 (1.64E-10–8.48E-9)	1 (<1–6)	<1 (<1–1)
COQ8B	p.Phe215Leufs*14	Eur	1/107,204	9.33E-06	8.70E-11 (5.58E-14–2.70E-9)	<1 (<1–1)	<1 (<1 –<1)
	p.Phe215Leufs*14	Eur	7/122,270	5.73E-05	3.28E-9 (5.30E-10–1.39E-8)	2 (<1–10)	<1 (<1–2)
		EAs	1/18,840	5.31E-05	2.82E-9 (1.81E-12–8.74E-8)	4 (<1–140)	<1 (<1 –<1)
		Lat	1/34,350	2.91E-05	8.48E-10 (5.43E-13–2.63E-8)	<1 (<1–16)	<1 (<1–1)
		Oth	1/6,400	1.56E-04	—	—	—
	p.Asp286Gly	Fin	4/24,036	1.66E-04	2.77E-8 (2.06E-9–1.82E-7)	<1 (<1 –<1)	<1 (<1 –<1)
	p.Arg320Trp	Afr	2/15,262	1.31E-04	1.72E-8 (2.52E-10–2.24E-7)	20 (<1–265)	<1 (<1–9)
		SAs	2/30,780	6.50E-05	4.22E-9 (6.19E-11–5.51E-8)	7 (<1–100)	<1 (<1 –<1)
		Eur	1/111,380	8.98E-06	8.06E-11 (5.17E-14–2.50E-9)	<1 (<1–1)	<1 (<1 –<1)
	p.His400Glnfs*11	Eur	1/111,710	8.95E-06	8.01E-11 (5.14E-14–2.49E-9)	<1 (<1–1)	<1 (<1 –<1)
	p.Glu447Glyfs*10	Eur	6/108,260	5.54E-05	3.07E-9 (4.14E-10–1.46E-8)	2 (<1–10)	<1 (<1–2)
	p.Glu483*	SAs	10/29,404	3.40E-04	1.16E-7 (2.66E-8–3.91E-7)	210 (48–712)	<1 (<1–1)
		Eur	1/101,248	9.88E-06	9.75E-11 (6.25E-14–3.03E-9)	<1 (<1–2)	<1 (<1 –<1)
COQ9	c.521 + 1delG	Eur	1/111,218	8.99E-06	8.08E-11 (5.18E-14–2.51E-9)	<1 (<1–1)	<1 (<1 –<1)
	p.Arg244*	Fin	2/22,296	8.97E-05	8.05E-9 (1.18E-10–1.05E-7)	<1 (<1 –<1)	<1 (<1 –<1)
		SAs	1/30,782	3.25E-05	1.06E-9 (6.76E-13–3.28E-8)	1 (<1–59)	<1 (<1 –<1)
Totals:						1,016 (200–6956)	122 (49–444)

^1^ **Ash**kenazi Jewish, **Afr**ican/African American, **Eur**opean (non-Finnish), **E**ast **As**ian, **S**outh **As**ian, **Lat**in American (admixed American), **Fin**nish, **Oth**er.

^2^Predicted homozygosity assuming Hardy-Weinberg equilibrium.

^3^Predicted number of homozygotes in population size equivalent to that of the given ethnicity globally.

^4^Predicted number of homozygotes in a population size equivalent to that the given ethnicity in the USA.

The presence of multiple variants within the same populations is consistent with the numerous reports of compound heterozygosity in patients with primary UQ deficiency^7^. When estimating birth prevalence of compound heterozygosity, pathogenic variants in COQ8A again exhibited a greater prevalence relative to other genes. In fact, the birth prevalence of compound heterozygotes for COQ8A among Ashkenazi Jews (1/725,578), Finns (1/1.4 M) or non-Finnish Europeans (1/1.6 M) alone were all individually greater than the combined prevalence of all other genes (1/17.1 M) (Table 3, full variant-by-variant breakdown in Table S3). We can estimate that 649 individuals worldwide are born as compound heterozygous for pathogenic genetic variants causing UQ deficiency, with 70 in the USA-like population.

**Table 3:** The predicted occurrence of compound heterozygotes of known pathogenic variants.

Gene	Pop^n^	Unique Genotypes	Summed Frequency^1^	Afflicted Worldwide	Afflicted USA
PDSS2	Eur	3	4.23E-09	2	0
	**All**	**3**	**5.28E-10**	**2**	**0**
COQ2	Afr	1	2.51E-09	2	0
	EAs	1	4.88E-09	7	0
	Eur	21	3.81E-08	21	3
	Lat	1	4.40E-09	2	0
	**All**	**22**	**6.24E-09**	**32**	**3**
COQ4	Eur	3	6.64E-09	3	0
	Lat	1	6.14E-09	3	0
	Oth	1	1.41E-07	—	—
	**All**	**3**	**1.92E-08**	**6**	**0**
COQ6	Afr	1	2.60E-08	30	1
	EAs	1	5.62E-09	9	0
	**All**	**1**	**3.95E-09**	**39**	**1**
COQ8A	Afr	3	7.19E-09	8	0
	Ash	6	1.38E-06	11	6
	EAs	1	3.38E-08	54	0
	Eur	210	6.20E-07	357	60
	Fin	3	7.02E-07	3	0
	Lat	21	9.03E-08	47	0
	SAs	10	3.06E-08	48	0
	Oth	21	8.58E-07	—	—
	**All**	**216**	**4.65E-07**	**528**	**66**
COQ8B	Eur	15	7.87E-09	2	0
	SAs	1	2.21E-08	40	0
	**All**	**15**	**3.75E-09**	**42**	**0**
Totals:		**259**	**4.99E-07**	**649**	**70**

See Table S3 for full variant-by-variant breakdown with confidence intervals.

^1^Subtotals (“All”) represent the average across all 8 populations, and the Total is the sum of those subtotals.

Premature stop codons, frameshifts or the disruption of canonical splice sites (LoF) or critical protein residues (via missense mutations) are all expected to result in significant impairments to protein function. Although we can expect an unknown proportion of these predicted pathogenic variants to result in embryonic lethality, those that do allow survival to birth are likely to result in clinically significant illness. We therefore determined the birth prevalence of all predicted pathogenic variants in UQ biosynthesis genes, as described in Methods. Across all UQ biosynthesis genes there were a total of 782 predicted pathogenic variants (including all known pathogenic variants), and 618 possible compound heterozygote combinations (summarized in Table 4, complete variant list in Table S4 and Table S5). The two genes with the highest frequency of predicted pathogenic variants (combining homozygotes and compound heterozygotes) were COQ8A and COQ8B, with cross-population average incidences of 1/193,621 and 1/198,391, resulting in a predicted 27,321 and 44,727 afflicted individuals worldwide, respectively, and 391 and 398 afflicted individuals in the USA. The gene with the lowest frequency was COQ3 (1/57 M), with only 146 predicted affected individuals worldwide, and none predicted in the USA. The population with the greatest total frequency of pathogenic variants was that of East Asia (1/20,170), with a predicted 79,423 afflicted individuals worldwide. The variant with the greatest prevalence in any population was COQ4 p.Arg240Cys in the Ashkenazi Jewish population, with a MAF of 0.0001719 (Table S4).

**Table 4:** Predicted prevalence of homozygous and compound heterozygous afflicted individuals for all known and predicted pathogenic variants.

Gene	Pop^n^	Homozygous				Compound Heterozygous			
		Unique Genotypes	Summed Frequency^1^	Afflicted Worldwide	Afflicted USA	Unique Genotypes	Summed Frequency^1^	Afflicted Worldwide	Afflicted USA
PDSS1	Afr	10	6.68E-08	77	<1	45	2.69E-07	297	<1
	Ash	2	2.06E-08	<1	<1	1	1.03E-08	<1	<1
	EAs	8	7.81E-07	1,256	4	28	8.60E-07	1,372	2
	Eur	33	2.46E-08	12	2	528	1.70E-07	27	1
	Fin	3	2.21E-08	<1	<1	3	1.41E-08	<1	<1
	Lat	11	1.53E-06	963	84	55	1.06E-06	643	50
	SAs	7	4.44E-08	76	<1	21	8.13E-08	132	<1
	Oth	3	9.98E-08	—	-	3	9.98E-08	—	—
	**All**	**62**	**3.23E-07**	**2,384**	**90**	**674**	**3.21E-07**	**2,471**	**53**
PDSS2	Afr	12	5.14E-07	607	18	66	9.00E-07	1,038	17
	Ash	1	2.03E-06	20	10	0	—	—	—
	EAs	13	5.03E-06	8,100	29	78	2.22E-06	3,532	3
	Eur	37	2.14E-08	9	1	666	2.15E-07	27	<1
	Lat	6	7.94E-09	2	<1	15	1.76E-08	5	<1
	SAs	12	1.74E-07	309	<1	66	4.20E-07	721	<1
	Oth	5	2.47E-07	—	—	10	4.16E-07	—	—
	**All**	**68**	**1.00E-06**	**9,047**	**58**	**879**	**5.23E-07**	**5,323**	**20**
COQ2	Afr	10	1.55E-07	180	2	45	5.53E-07	628	6
	Ash	1	1.70E-06	17	8	0	—	—	—
	EAs	5	4.01E-07	644	2	10	1.99E-07	315	<1
	Eur	31	7.51E-08	48	11	465	2.99E-07	106	12
	Fin	2	2.24E-08	<1	<1	1	8.18E-09	<1	<1
	Lat	7	4.53E-08	26	1	21	9.31E-08	47	<1
	SAs	10	1.86E-08	29	0	45	7.24E-08	119	<1
	Oth	3	1.10E-06	—	—	3	3.80E-07	—	—
	**All**	**53**	**4.40E-07**	**944**	**24**	**573**	**2.00E-07**	**1,215**	**18**
COQ3	Afr	2	4.51E-08	53	1	1	8.67E-09	10	<1
	EAs	3	2.10E-08	33	<<1	3	1.72E-08	27	<1
	Eur	16	7.30E-09	3	<1	120	2.62E-08	1	<1
	Lat	1	3.55E-09	2	<1	0	—	—	—
	SAs	4	4.75E-09	6	<1	6	7.09E-09	11	<1
	**All**	**23**	**1.02E-08**	**97**	**1**	**130**	**7.40E-09**	**49**	<**1**
COQ4	Afr	19	2.22E-07	257	2	171	1.60E-06	1,811	5
	Ash	1	9.55E-06	95	48	0	—	—	—
	EAs	11	2.61E-06	4,195	13	55	2.60E-06	4,169	7
	Eur	40	5.10E-08	29	4	780	4.90E-07	129	8
	Fin	5	1.11E-06	5	<1	10	2.01E-07	<1	<1
	Lat	17	8.40E-08	44	2	136	4.04E-07	187	2
	SAs	17	3.19E-08	45	<1	136	2.30E-07	334	<1
	Oth	9	1.87E-06	—	—	36	3.61E-06	—	—
	**All**	**81**	**1.94E-06**	**4,670**	**69**	**1,236**	**1.14E-06**	**6,630**	**22**
COQ5	Afr	4	1.71E-08	20	<1	6	2.56E-08	30	<1
	EAs	5	5.11E-07	822	2	10	3.92E-07	627	1
	Eur	11	1.32E-08	6	<1	55	4.02E-08	11	<1
	Fin	1	2.01E-09	<1	<1	0	—	—	—
	Lat	2	1.77E-09	<1	<1	1	8.87E-10	<1	<1
	SAs	1	1.06E-09	1	<1	0	—	—	—
	**All**	**23**	**6.83E-08**	**849**	**2**	**72**	**5.74E-08**	**668**	**1**
COQ6	Afr	20	2.32E-07	270	2	190	1.79E-06	2,045	6
	Ash	3	3.09E-08	<1	<1	3	3.09E-08	<1	<1
	EAs	18	1.75E-07	274	<1	153	1.08E-06	1,642	<1
	Eur	58	1.26E-07	80	17	1,653	1.06E-06	385	48
	Fin	4	7.03E-09	<1	<1	6	1.05E-08	<1	<1
	Lat	18	1.53E-06	962	83	153	1.66E-06	984	67
	SAs	21	3.06E-07	540	<1	210	6.91E-07	1,076	<1
	Oth	9	1.52E-06	—	—	36	3.40E-06	—	—
	**All**	**110**	**4.91E-07**	**2,126**	**102**	**2,284**	**1.22E-06**	**6,132**	**121**
COQ7	Afr	2	1.38E-08	16	<1	1	6.92E-09	8	<1
	EAs	2	6.96E-09	10	<1	1	3.48E-09	5	<1
	Eur	10	8.85E-09	5	<1	45	1.90E-08	3	<1
	Fin	2	4.46E-07	2	<1	1	5.14E-08	<1	<1
	Lat	1	8.87E-10	<1	<1	0	—	—	—
	SAs	5	3.84E-08	66	<1	10	4.99E-08	85	<1
	Oth	2	5.72E-08	—	—	1	2.82E-08	—	—
	**All**	**18**	**7.15E-08**	**99**	<**1**	**58**	**1.99E-08**	**101**	<**1**
ADCK3	Afr	23	3.16E-07	369	7	253	2.39E-06	2,736	19
	Ash	4	7.08E-06	69	35	6	1.38E-06	11	6
	EAs	17	2.47E-06	3,970	12	136	6.50E-06	10,392	15
	Eur	97	1.99E-07	120	23	4,656	3.81E-06	1,437	154
	Fin	12	1.77E-06	7	<1	66	3.95E-06	11	<1
	Lat	26	8.76E-07	541	45	325	2.41E-06	1,361	72
	SAs	34	1.23E-06	2,209	3	561	2.47E-06	4,088	<1
	Oth	14	6.57E-07	—	—	91	3.82E-06	—	—
	**All**	**149**	**1.82E-06**	**7,285**	**125**	**5,635**	**3.34E-06**	**20,036**	**266**
ADCK4	Afr	34	8.77E-07	1,031	26	561	6.97E-06	8,043	109
	Ash	6	3.37E-06	32	16	15	1.74E-06	11	3
	EAs	24	3.70E-06	5,945	18	276	1.63E-05	26,171	44
	Eur	83	1.34E-07	79	14	3,403	2.20E-06	725	74
	Fin	11	1.54E-07	<1	<1	55	4.68E-07	<1	<1
	Lat	30	5.79E-07	352	27	435	2.43E-06	1,349	67
	SAs	17	1.51E-07	262	<1	136	4.54E-07	727	<1
	Oth	6	2.38E-07	—	—	15	5.40E-07	—	—
	**All**	**135**	**1.15E-06**	**7,701**	**101**	**4,449**	**3.89E-06**	**37,026**	**297**
COQ9	Afr	9	8.83E-07	1,045	33	36	1.19E-06	1,401	38
	EAs	10	1.71E-06	2,751	9	45	1.98E-06	3,171	4
	Eur	34	1.82E-08	7	1	561	1.59E-07	18	<1
	Fin	5	1.62E-07	<1	<1	10	1.74E-07	<1	<1
	Lat	15	1.06E-07	59	3	105	4.28E-07	221	4
	SAs	11	4.34E-08	70	<1	55	1.31E-07	193	<1
	Oth	3	1.55E-07	—	—	3	1.34E-07	—	—
	**All**	**60**	**3.85E-07**	**3,932**	**46**	**768**	**5.25E-07**	**5,004**	**46**
Totals:		**782**	**7.70E-06**	**39,134**	**618**	**16,758**	**1.12E-05**	**84,655**	**844**

See Tables S4 and S5 for full variant-by-variant breakdowns with confidence intervals.

^1^Frequencies for each population are the sum of all homozygous or compound heterozygote frequencies for each variant in that population; Subtotals for all populations are the average of the 8 populations considered, and Total is the sum of those subtotals.

Considering the occurrence of both homozygotes and compound heterozygotes averaged across all populations, our results predict a global birth prevalence of 1/52,092. However, not all the populations considered are of equal size, and the predicted number of afflicted individuals worldwide was 41,555 due to homozygosity and 85,581 due to compound heterozygosity, for a total of 123,789 (1/48,495). In the USA, our analysis predicts 1,462 afflicted individuals (1/211,917).

## Discussion

**Figure 1:**
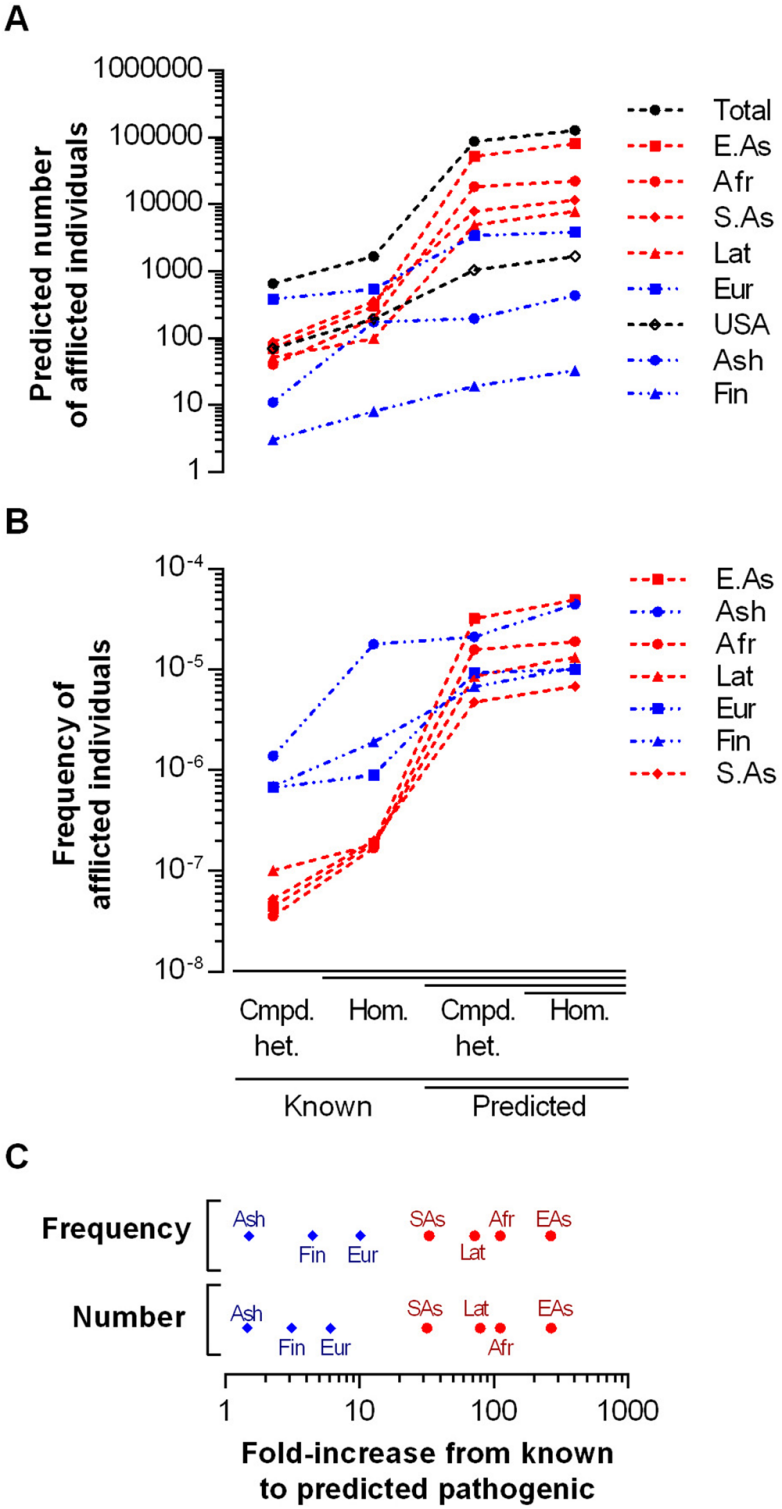
Prevalence of primary UQ deficiency based on known and predicted pathogenic variants. (**A**) Predicted number of afflicted individuals due to compound heterozygosity or homozygosity of known or predicted pathogenic variants, as denoted on x-axis, for each population. (**B**) Frequency of afflicted individuals within each analyzed population. (**C**) Contribution of known or predicted pathogenic variants to the frequency or number of afflicted individuals within each population. The fold-difference between known pathogenic variants only, and the total of known and predicted pathogenic variants, is shown on the x-axis.

Overall, our results predict a worldwide total of 123,789 individuals suffering from primary UQ deficiency, and 1,462 in a population with a composition similar to the USA. Of these, 1,665 and 192 respectively are due to variants that are known to be pathogenic, with the remainder due to predicted LoF and pathogenic missense variants (summarized in Fig. 1A and B). However, the extent to which known pathogenic variants contributed to the total varied between populations. The addition of predicted LoF variants has less impact for Western populations (Ashkenazi Jews, Finnish and non-Finish Europeans: blue in Fig. 1), with inclusion of predicted pathogenic variants resulting in an average 3.5-fold increase in the number of afflicted individuals, relative to known pathogenic variants only (Fig. 1c). In contrast, in populations from non-Western, developing regions (South and East Asians, Latin Americans and Africans: red in Fig. 1), the addition of predicted LoF variants resulted in an average 122-fold increase in the number of afflicted individuals (Fig. 1c). The increased likelihood of pathogenic variants to have been identified in Western populations is consistent with the reality of their relatively higher clinical coverage compared to non-Western populations, where the expense of clinical sequencing has limited the genetic characterization of patients suffering from mitochondrial disease. Our results imply that primary UQ deficiency is substantially under-diagnosed in Latin American, African and Asian populations.

There are several factors that could induce error in our predictions. For example, LoF variants may be so harmful that a homozygous individual is not viable in the first place. That this is possible is supported by the embryonic lethality of the complete genetic ablation of PDSS2, COQ2, COQ3, COQ6 and COQ7 in mice^16–19^, with COQ4 exhibiting pre-weaning lethality^17^. In contrast, COQ8A^23^ and COQ9^24^ –null mice have been reported as viable. Indeed, among patients with pathogenic variants in the UQ biosynthesis genes likely to be necessary for life (PDSS1 – COQ7), very few are homozygous or compound heterozygous for severe variants expected to result in significant LoF. Among the severe variants (nonsense, frameshift, splice site affecting) for these genes described in the literature we reviewed, only COQ2 p.Asn401Ilefs*15 was present in homozygous form, resulting in multi-organ failure and death in an infant patient^25^, and there was only one patient compound heterozygous for LoF variants (COQ6 p.Trp447* and p.Gln461fs*478^26^). In all three variants the region affected was close to the C-terminus (closer than any other known pathogenic variant for these genes), implying that these patients may have retained some partially functional protein, and that other severe variants may have resulted in complete LoF and embryonic lethality.

It is therefore likely that some of the LoF variants that contribute to our final totals may not actually contribute to disease rates due to embryonic or pre-natal lethality. Variant severity is not easy to predict – for example, COQ9^R239X^ mice that express a partial protein have a much more severe phenotype than COQ9^Q95X^ mice with no measurable protein expression, presumably due to the destabilization of a multiprotein UQ biosynthesis complex by the truncated protein^27^. However, homozygous or compound heterozygous severe variants in PDSS1 through COQ7 account for only 6,142 out of 123,789 predicted individuals worldwide, and 219 out 1,462 in the US. This suggests that our predictions are not greatly inflated by the inclusion of embryonically lethal allelic combinations.

Our predictions may also suffer from the opposite problem - missense variants identified as damaging by SIFT or PolyPhen2 may, in fact, not have deleterious physiological effects. We attempted to address this by requiring our “predicted pathogenic” variants to be rated as highly likely to be deleterious by both PolyPhen2 and SIFT, but such prediction algorithms are clearly not infallible. For example, COQ4 p.Arg145Gly was rated as “tolerated” by SIFT, yet was reported in homozygous form in a neonate who died 4 h after birth, and it also failed to rescue Δcoq4 yeast^28^. It is therefore reasonable to expect a certain proportion of predicted-pathogenic missense variants to result in asymptomatic individuals. Interestingly, missense variants seem to be responsible for a lesser proportion of COQ8A and COQ8B-deficient individuals, with patients homozygous or compound heterozygous for LoF variants being relatively common^29–32^. Given that COQ8A alone can rescue COQ8-null yeast^33^, and COQ8A patients with truncating nonsense mutations shown to result in nonsense-mediated decay remained viable in their mid-20’s^32^, it is likely that these genes may be relatively insensitive to some borderline-pathogenic missense variants. This has the potential to greatly impact our predictions, with homozygous or compound heterozygous variants in COQ8A and COQ9B accounting for 46,654 out of 123,789 predicted affected individuals worldwide, and 559 out of 1,462 in the USA.

COQ8A and COQ8B are also noteworthy in that most of the known patients have relatively well-defined, gene-specific, pathologies. Specifically, symptoms of ataxia (often associated with cerebellar atrophy or other neurological abnormalities) are found with 26 of the 29 known pathogenic variants of COQ8A, and all 13 of the published COQ8B pathogenic variants exhibited nephrotic syndrome (citations provided in Table S2). It would therefore be tempting to claim that our predicted patients would exhibit similar clinical conditions, with, for example, all predicted COQ8B patients suffering from nephrotic syndrome^34^. However, it is likely (and our results support) that only a subset of primary UQ deficiency patients have been identified at this point, and they may be non-representative of the actual patient population. Of note, many of the known pathogenic variants were identified in studies where clinicians screened cohorts of patients with specific subsets of well-defined symptoms. For example, our knowledge of COQ8B variants largely comes from two studies in which large numbers of patients with nephrotic syndrome were subjected to sequencing of either whole exomes or multi-gene panels designed for nephrotic syndrome^29,35^. A similar issue can be raised for the ataxic nature of COQ8A variants. For example, two studies described how, after identifying pathogenic COQ8A variants in ataxic patients, they proceeded to sequence COQ8A in other ataxic patients, identifying additional novel pathogenic variants^30,32^. Additional pathogenic variants were found in later studies in which COQ8A, alone or in combination with other UQ biosynthesis genes, was specifically sequenced in ataxic patients^31,36^. We hypothesize that future COQ8A or COQ8B patients identified via less targeted methodologies may present with more diverse clinical phenotypes, as is characteristic of other UQ biosynthesis genes such as COQ2 or COQ4.

There are also several factors that could increase the number of afflicted individuals beyond our estimates. For example, we conservatively assumed that primary UQ deficiency is always recessive; however, haploinsufficiency of COQ4 has been shown to cause clinically significant primary UQ deficiency^37^. Also, violations of Hardy-Weinberg equilibrium (e.g., consanguinity or populations with a large degree of endogamy) could increase the likelihood of an individual being born with two pathogenic variants. It is also noteworthy that 6 of the 29 missense variants known to be pathogenic would not have met our criteria for inclusion as “predicted” pathogenic variants, since they were not assigned the highest level of confidence for pathogenicity by both SIFT and PolyPhen2 (Table 1). This supports the conservative nature of our selection criteria.

Furthermore, there are several reasons why truly pathogenic variants may not appear on our list of known variants. Some variants may have been identified in clinics without being formally described in the literature. For example, COQ2 p.Met128Val and p.Arg387* have been cited as pathogenic in the secondary literature^38^, but without a formal research citation they would not have met our inclusion criteria as known pathogenic variants. Furthermore, although the latter variant was included as a predicted pathogenic variant, the former was assessed as ‘benign’ and ‘tolerated’ by Polyphen and SIFT respectively, excluding it from our list of predicted pathogenic variants. In addition, many predicted pathogenic variants were more common in non-western populations, meaning that they are less likely to have been identified in the existing clinical reports, which have focussed on western populations. Additionally, our list of known pathogenic variants may not have included variants detected as part of recent large-scale studies^39–41^, and the fact that some UQ biosynthesis genes were found to be associated with disease earlier than others (e.g., COQ2 was first found in 2006^42^, vs. COQ8B in 2013^29^ and COQ7 in 2015^43^) could have delayed the introduction of some genes into widely used genetic screening panels^44^, meaning that more patients were screened for some genes compared to others. Finally, after the literature review phase of our analysis was concluded, novel pathogenic variants have continued to be described in the clinical literature (e.g., COQ4^45^, COQ6^46^, COQ7^47^, ADCK4^48,49^), indicating that many remain to be reported.

Several aspects of our results point towards their general reliability. For example, there have been no reports of pathogenic variants in COQ3 or COQ5, which is consistent with our prediction of few individuals with primary UQ deficiency due to pathogenic variants in these genes (less than 2,000 individuals worldwide, and only 4 in the USA). Conversely, more patients with defects in COQ8A and COQ8B have been described than for any other UQ biosynthesis gene^8^, which corresponds to our finding that pathogenic variants in these genes make the greatest contribution to the number of individuals worldwide predicted to suffer from primary UQ deficiency, together accounting for more than half of the predicted 127,136 patients worldwide.

In conclusion, we have made the first estimates of the worldwide and within-population birth prevalence of individuals who are homozygous or compound heterozygous for pathogenic variants causing primary UQ deficiency by combining a decades-worth of clinical genetics with the recently available large-scale full exome/genome sequencing. Our calculations suggest a minimum of 1,665 afflicted individuals worldwide or 192 in the USA (using only variants clinically shown to be pathogenic), up to a maximum of 123,789 worldwide or 1,462 in the USA (with all variants predicted to be pathogenic). Notably, the gap between predictions made using “known” vs. “predicted” pathogenic variants appears smallest in populations expected to have the greatest access to the modern methodologies of clinical genetics. This implies that healthcare providers have already made substantial headway in identifying individuals suffering from this disorder. However, it remains likely that the bulk of patients worldwide suffering from primary UQ deficiency have yet to be recognized.

## Electronic supplementary material

Supplemental Tables

Figure S1

File S1
