# Supplementary material for: Estimating the occurrence of primary ubiquinone deficiency by analysis of large-scale sequencing data
Source: Sci Rep. 2017 Dec 18;7:17744. doi: 10.1038/s41598-017-17564-y (PMC5735152; doi:10.1038/s41598-017-17564-y)
Supplement: Supplementary file 2 — Figure S1 [file 41598_2017_17564_MOESM2_ESM.pdf]

Estimating the occurrence of primary ubiquinone deficiency by analysis of large-scale sequencing data

Supplemental Figure

Bryan G. Hughes[1], Paul M. Harrison[1] & Siegfried Hekimi[1]*

[1] Department of Biology, McGill University, Montreal, Canada

* Corresponding author: 1235 Avenue Docteur Penfield, H3A 1B1 Montreal, 514-398-6440
siegfried.hekimi@mcgill.ca

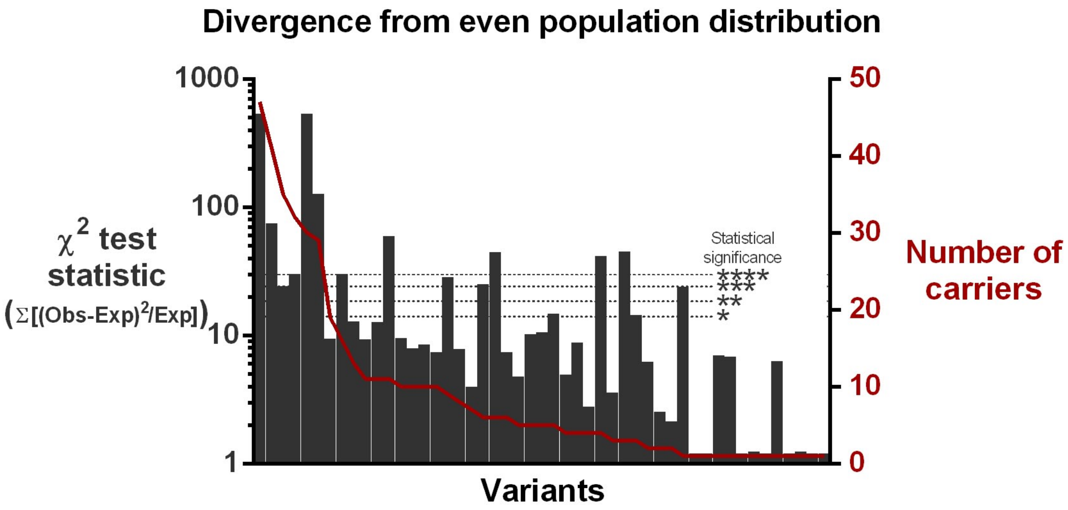

As a measure of divergence from an expected, even, distribution across populations, χ^2 test statistics (**dark grey**) of allele frequencies for each variant are plotted on the left *x*-axis. The number of carriers for each variant (**red**) is plotted on the right *x*-axis.

If variants are evenly distributed within populations (i.e., equal frequencies), then the difference between the observed and expected (based on assumption of equal frequencies) carrier numbers should be minimal and the χ^2 test statistic will be small. If a variant is present in one or more populations in a frequency much different than the other populations, then χ^2 will be larger. Variants with fewer carriers (i.e., a smaller sample size) should result in a χ^2 test with lower statistical power.
